# Place in Therapy of the Newly Available Armamentarium for Multi-Drug-Resistant Gram-Negative Pathogens: Proposal of a Prescription Algorithm

**DOI:** 10.3390/antibiotics10121475

**Published:** 2021-11-30

**Authors:** Lorenzo Volpicelli, Mario Venditti, Giancarlo Ceccarelli, Alessandra Oliva

**Affiliations:** Department of Public Health and Infectious Diseases, Sapienza University of Rome, 00185 Rome, Italy; lorenzo.volpicelli@uniroma1.it (L.V.); giancarlo.ceccarelli@uniroma1.it (G.C.); alessandra.oliva@uniroma1.it (A.O.)

**Keywords:** antimicrobial stewardship, ceftazidime/avibactam, meropenem/vaborbactam, cefiderocol, place in therapy, multidrug resistant organisms, carbapenemase-producing *Enterobacterales*, carbapenem-resistant *Acinetobacter baumannii*

## Abstract

The worldwide propagation of antimicrobial resistance represents one of the biggest threats to global health and development. Multi-drug-resistant organisms (MDROs), including carbapenem-resistant non-fermenting Gram-negatives and *Enterobacterales*, present a heterogeneous and mutating spread. Infections by MDRO are often associated with an unfavorable outcome, especially among critically ill populations. The polymyxins represented the backbone of antibiotic regimens for Gram-negative MDROs in recent decades, but their use presents multiple pitfalls. Luckily, new agents with potent activity against MDROs have become available in recent times and more are yet to come. Now, we have the duty to make the best use of these new therapeutic tools in order not to prematurely compromise their effectiveness and at the same time improve patients’ outcomes. We reviewed the current literature on ceftazidime/avibactam, meropenem/vaborbactam and cefiderocol, focusing on antimicrobial spectrum, on the prevalence and mechanisms of resistance development and on the main in vitro and clinical experiences available so far. Subsequently, we performed a step-by-step construction of a speculative algorithm for a reasoned prescription of these new antibiotics, contemplating both empirical and targeted use. Attention was specifically posed on patients with life-risk conditions and in settings with elevated prevalence of MDRO.

## 1. Introduction

In 2020, WHO outlined multi-drug-resistant organisms (MDROs) as one of the biggest threats to global health, development, and food security; this phenomenon is accelerated by misuse of antibiotics and produces longer hospital stays, higher medical costs and increased mortality [1]. The worldwide spread of resistances has not occurred uniformly, but shows regional and even local heterogeneity. As for carbapenemase enzymes, metallo-β-lactamases (MBLs) prevail in the Indian sub-continent and Balkan states, *Klebsiella pneumoniae* carbapenemase (KPC) in United States, Israel, Greece and Italy, and the oxacillinase-β-lactamases (OXA-48) in Turkey, the Middle East and North Africa. Nevertheless, the risk of the sudden introduction of a new MDRO into regions of non-endemicity via cross-border patient transfers or returning travelers is on the prowl, as witnessed by the two distinct outbreaks of New Delhi MBL (NDM) producing *K. pneumoniae* infections that occurred in Tuscany and Western Pomerania in 2019 [2,3].

In terms of deaths and disability adjusted life years (DALY) Italy has felt the greatest impact of MDRO in the European Economic Area (EEA) [4]. Even considering its ageing population, one third of MDRO-related deaths of the EEA occur in Italy, heavily affecting infants and older people. This burden is mostly healthcare-associated and comparable to the cumulative effect of influenza, tuberculosis and HIV [4]. After the adoption in 2017 of the National Action Plan on Antimicrobial Resistance (PNCAR), Italian antibiotic consumption is now slowly decreasing, although with regional discrepancies and an overall utilization still higher than the European average [5]. National plans are probably not enough, and some experts are now demanding a decisive intervention of the European Parliament in a similar manner to that successfully used to fight air pollution [6].

Infections from MDRO are generally associated with poor prognosis. A retrospective study on Gram-negative (GN) bloodstream infections (BSIs) in 173 US hospitals defined difficult-to-treat resistance (DTR) as non-susceptibility to all first-line agents, which severely constrains treatment options. The risk of death significantly increased as the number of active first-line agents fell to zero [7]. Infections by *A. baumannii* with DTR (DTR-*Ab*), which exhibits resistance to all β-lactams, β-lactam/β-lactamases inhibitor combinations and fluoroquinolones, present an increased fatality of over 40% [8]. In countries with endemic presence of MDRO pathogens like Italy, highly fatal outbreaks frequently occur, especially among critically ill patients in intensive care units (ICUs) [9,10]. Data collected during a 10-year period within the Italian SPIN-UTI network showed that 6.2% of patients admitted to ICU developed sepsis, with a case fatality rate of 46%. *Acinetobacter baumannii*, *Klebsiella pneumoniae* and *Pseudomonas aeruginosa* accounted for 44.9% of isolates [11].

Effective prevention and management of outbreaks, together with multidisciplinary, shared and finalized antimicrobial stewardship (AMS) programs are of paramount importance to contain MDRO-related morbidity and mortality, especially in ICU settings [12]. In recent decades, polymyxins gained renewed interest in the management of MDRO as they demonstrated potent activity against carbapenem-resistant (CR) GNs. Limitations in their clinical use are imposed by the outstanding incidence of renal toxicity, their poor lung, bone and central nervous system penetration and the lack of an accurate and practical susceptibility testing. Nowadays, the rising trend of resistance requires new molecules with the potential to act as colistin-supplanting agents [8,13].

Ceftazidime/avibactam (CZA), meropenem/vaborbactam (MVB) and cefiderocol (CFDC) are three recently introduced antimicrobials. Taken as a whole, these drugs represent a hopeful answer to the top tier global priority list of MDRO published by WHO: CR *A. baumannii*, CR *P. aeruginosa* and CR, third generation cephalosporin-resistant *Enterobacterales* [14,15].

Here, we firstly revise the current literature highlighting the pearls, pitfalls, and upcoming features of CZA, MVB and CFDC. Secondly, we propose and describe a simple clinical and epidemiology-based algorithm that speculatively outlines a reasoned utilization of those latecomer and probably “game changing” antibiotics.

While imipenem/relebactam and plazomicin have only recently entered the market, many new promising molecules including cefepime/vaborbactam and sulbactam/durlobactam are still in a phase three trial. Therefore, we focused our attention on CZA, MVB and CFDC rather than other new antibiotics in the pipeline as they have been available for longer and benefit from a wider available literature and real-life clinical experience.

## 2. Results

### 2.1. Ceftazidime/Avibactam

Ceftazidime/avibactam (CZA) is a second-generation intravenous β-lactam/β-lactamase inhibitor combination composed of avibactam, a reversible diazabicyclooctane inhibitor of class A, class C and some class D β-lactamases, coupled with ceftazidime, a third-generation anti-*Pseudomonas* cephalosporin in a 4:1 ratio [16,17]. In vitro studies have shown that avibactam, through covalent acylation of its targets, restores the activity of ceftazidime against extended-spectrum beta-lactamase (ESBL), AmpC, KPC, OXA-48 producing *Enterobacterales* and *P. aeruginosa* with DTR (DTR-*Pa*), including strains producing GES carbapenemase. Despite this broad antimicrobial spectrum, CZA has no activity on MBLs and most *Acinetobacter spp*. isolates [18] (Table 1).

The European Medicines Agency (EMA) approved CZA for complicated intrabdominal (cIAI) and urinary tract (cUTI) infections, hospital-acquired (HAP) and ventilator-associated (VAP) pneumonia and for GN infections with limited treatment options [19]. In the pre-CZA era, infections by CR *Enterobacterales* (CRE) were burdened by a 30–50% mortality rate [20]. In a meta-analysis including 54 studies conducted up until 2018 and 3352 patients with CR *K. pneumoniae* infection, pooled mortality was 37.2%, with no significant difference between carbapenem-, polymyxin-, aminoglycoside- and tigecycline-containing or other carbapenem-sparing regimens [21]. Therefore, after its marketing authorization, CZA was quickly established as treatment of choice. In a retrospective observational study on 138 adults with KPC-producing *K. pneumoniae* (KPC-*Kp*) infection that received CZA as salvage therapy after a first-line treatment, the mortality rate was significantly lower respect to the control group (36.5% vs. 55.8%), both in those receiving a single drug and a combination regimen [22]. Among mechanically ventilated patients with life-threatening infections caused by CRE, mostly with septic shock and multiorgan failure, a CZA-containing regimen was an independent predictor of survival and clinical cure [23]. Recently, Karaiskos et al. found a significantly lower mortality in subjects with KPC-*Kp* BSI treated with a CZA-containing regimen with respect to those treated with “classic” agents, mainly colistin and tigecycline (18.3% vs. 40.8%) [24]. CZA has been confirmed to be a valuable treatment option for KPC-*Kp* in a large retrospective analysis of real-life post marketing CZA use in 22 Italian hospitals. Of note, mortality was negatively associated with CZA administered as prolonged infusion (≥3 h). Despite the fact that over 70% of cases were managed through association regimens (usually with fosfomycin, tigecycline, gentamicin and meropenem) authors found no significant difference in mortality between CZA alone or in combination, whereas favorable trends were observed among patients with VAP [25]. Although evidence is limited for infections due to OXA-48 producing *Enterobacterales*, avibactam showed strong inhibitory properties against OXA-48 and significantly reduced MICs of ceftazidime, cefepime and imipenem [26]. CZA was demonstrated to be effective and have a better safety profile compared to the best available therapy (BAT) in OXA-48 infections; results were comparable to those observed in *Enterobacterales* infections due to KPC producers [27]. Hirsh and colleagues tried out CZA and ceftolozane/tazobactam against a collection of *P. aeruginosa*, obtaining outstanding results: both drugs were active in >80% of β-lactam resistant isolates (75% meropenem non-susceptible) and on 88% of DTR-*Pa* [28]. In an investigation in 360 *P. aeruginosa* strains, CZA was the most active compound against meropenem- and imipenem-resistant strains (92.6% and 93.8% susceptible, respectively) [29]. The antimicrobial activity of CZA and comparator agents was also assessed against a collection of 8615 isolates of *Enterobacterales* and *P. aeruginosa* from central European countries and Israel. CZA susceptibility rate was high and stable in the two time periods considered (2014–2017 and 2018), although alarming trends of resistance to comparators were detected [30]. The importance of CZA in the management of *Pseudomonas* infections was further increased by the voluntary manufacturing stop and global product recall of ceftolozane/tazobactam that Merk conducted from December 2020, because of compromised sterility process [31]. Despite CZA displaying no activity on MBL-producing *Enterobacterales*, the combination of CZA with aztreonam showed promising results in a recent observational multicenter study where colistin-based regimens had a significantly higher mortality [32]. In a prospective pharmacokinetic (PK) study, standard dosing of triple combination CZA + avibactam was investigated in a complex population of critically ill patients with multiple comorbidities. Estimated kidney function through CKD-EPI equation represented the primary covariate influencing PK; this result was probably largely driven by the elderly population included [33].

As for *Stenotrophomonas maltophilia*, one of the leading MDROs that urgently needs new treatment options, Lin et al. recently published an in vitro study on 76 non-repetitive strains. Results were promising, as the addition of avibactam to ceftazidime and aztreonam restored susceptibility to ∼50% and ∼90% of a collection of isolates, respectively [34]. Of note, in this study, approximately 80% of tested isolates were selected for their non susceptibility to minocycline, levofloxacin and cotrimoxazole [34].

According to European, U.S. and Asian reports, the baseline resistance rate to CZA in *Enterobacterales* is less than 2.6% but raises to over 8% in DTR-*Pa* strains and even 74% in strains of *A. baumannii* from ICU [35]. A systematic literature review found 80% of KPC-*Kp*-resistant isolates were reported in the U.S.A., Greece, and Italy, mostly belonging to ST258 strain and one third with no previous exposure to CZA. Restoration of meropenem susceptibility occurred in 52.6% of isolates with CZA resistance acquired during treatment [36]. The main resistance mechanism is represented by point mutations that increase the flexibility in the Ω-loop of lactamase, enhancing its ability to trap ceftazidime and decreasing the binding of avibactam [17]. The accumulation of multiple resistance mechanisms is probably responsible for CZA resistance, also involving mutations in Omp35 and Omp36 porin channels and efflux pumps overexpression [35]. Very recently, Carattoli et al. characterized nine different CZA-resistant KPC-3 variants, five of them never previously reported, after a one-year period of microbiologic surveillance. Some isolates retained carbapenem-susceptibility with meropenem MIC <8 mg/L. Worryingly, the vertical evolutionary trajectory of some clones as well as the transmission of CZA-resistant strains to untreated patients was observed [37].

Real-life studies disclosed a CZA treatment failure rate of around 10–30% when used on susceptible CR pathogens [38,39]. Some clinical situations have been associated to the risk of in vivo resistance to CZA, probably through an under-exposure effect resulting in a deranged PK/pharmacodynamic (PD) profile [40]. In 2015, Nicolau et al. demonstrated a dose-proportional ELF penetration of only 30% compared to plasma levels in healthy volunteers [41] and comparable results were observed in infected neutropenic mice [42]. Shields et al. found that pneumonia was an independent risk factor for CZA clinical failure, while the receipt of renal replacement therapy (RRT) predicted microbiological failure and CZA resistance development [39]. Recently, clinical and microbiological failures with CZA have combined to the development of decreased susceptibility/resistance to this agent, as described in a case of septic thrombosis, a high clinical complexity condition [43], and in a case of delayed source control [44].

**Figure 1:**
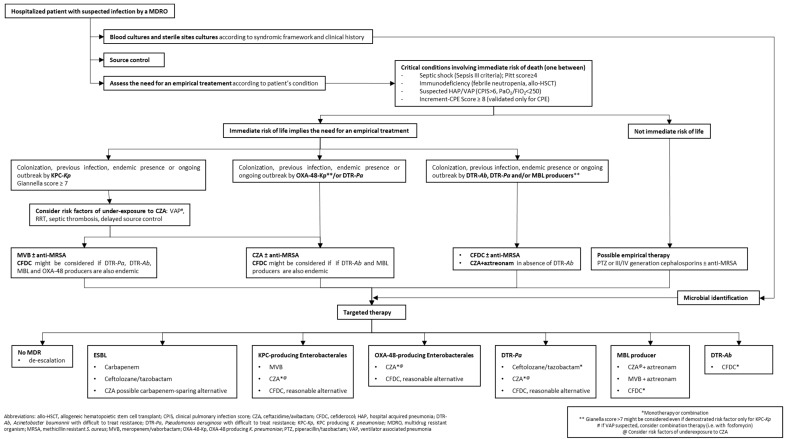
Algorithm for a reasoned prescription of new antimicrobials.

Given its clinical and microbiological characteristics, CZA should be considered the preferred agent not only in settings with a predominance of KPC-producing organisms, but also in settings with OXA-48 or with OXA-48/KPC co-prevalence [45] (Figure 1).

### 2.2. Meropenem/Vaborbactam

Meropenem/vaborbactam (MVB) is a recently approved coformulation where vaborbactam, a new generation, competitive inhibitor with a boron-ring structure and high affinity to serine-β-lactamase, preserves the activity of meropenem, a carbapenem with broad spectrum and anti-*Pseudomonas* action, on KPC-producing *Enterobacterales* [46]. Castanheira et al. tested MVB against 14,304 worldwide collected GN isolates and 99.6% of *Enterobacterales* were inhibited. Considering only CR strains, MVB was the most active β-lactam agent (84.2%), with lower MIC_50_/MIC_90_ values in US and Latin American isolates and higher in those from the Asian Pacific region. Unfortunately, the activity of MVB was similar to that of meropenem alone on *P. aeruginosa*, *A. baumannii* and *S. maltophilia* [47]. No susceptibility of MVB was showed against strains producing OXA-48 carbapenemase, and only weak inhibition activity of vaborbactam was evoked on MBL enzymes [48] (Table 1). An in vitro study tested MVB and comparators against a global collection of 991 KPC-positive *Enterobacterales* and susceptibility was obtained in 99%. Notably, based on MIC_90_ values, MVB was 4 times and 32 times more potent than CZA on *K. pneumoniae* and *E. coli*, respectively. No difference emerged when stratified by KPC variant type [49]. This new combination benefits of optimal PK properties: the PK profiles of the two molecules are virtually superimposable, with vaborbactam cleared slightly slower to effectively protect meropenem. This allows a high-dose, extended-infusion regimen that minimizes the development of resistance and achieves identical concentration-time profiles in extravascular compartments such as epithelial lining fluid (ELF) [46]. The TANGO I trial compared this first carbapenem/β-lactamase inhibitor combination with piperacillin/tazobactam (PTZ) for cUTI and pyelonephritis. Non-inferiority was demonstrated, although 15% of isolates tested resistant to PTZ [50]. The TANGO II evaluated efficacy and safety of MVB monotherapy versus BAT on patients with a CRE infection and showed an increased clinical cure and decreased mortality and nephrotoxicity [51]. Later, a post-hoc analysis of TANGO II conducted on 38 subjects with no prior antimicrobial failure found MVB superior to BAT. The authors suggested an advantage of MVB when administered as first-line and raised the question about finding a correct balance between curtailing the use of novel agents and simultaneously offering active early therapy, especially for difficult-to-treat vulnerable patients [52]. In Europe, MVB has been approved for cIAI, cUTI, HAP and VAP, for BSI associated with one of those conditions and for GN infections in patients with limited treatment options [53].

As described above, CZA-resistance selection is mainly linked to acquired structural changes in the KPC enzyme. Meanwhile, it has been shown that MVB-resistance is primarily driven by an increase of *bla*_KPC_ copy number and by porin loss/mutations [17,54]. Importantly, these resistance mechanisms can be prevented by the drug concentrations achieved with an optimal dosing. In other words, MVB detains a higher barrier to resistance to the KPC Ω-loop binding site mutations, with D179Y being the most frequently associated with CZA-resistance development [54]. Direct clinical comparison of CZA and MVB on KPC-producing *Enterobacterales* is still lacking. However, Ackley et al. performed a retrospective analysis comparing CZA monotherapy, CZA combination and MVB monotherapy. Despite similar clinical success and adverse event rates, more patients in the CZA monotherapy arm underwent recurrent infection, five had increased MIC of CZA and three developed CZA resistance. As stated by the authors, it is intriguing to note that all the three patients had respiratory source of infection and received RRT [55].

MVB should probably be considered the preferred agent against organisms with confirmed production of KPC2 and KPC3 enzymes due to its enhanced in vitro potency and reduced emergence of resistance [45]. In the setting of empirical treatment, MVB should be taken into consideration in contexts of KPC predominance over other mechanism of carbapenem resistance [56]. This is particularly true in the aforementioned situations that underpin a risk of CZA under-exposure (severe pneumonia, RRT, septic thrombosis and delayed source control) (Figure 1).

### 2.3. Cefiderocol

The advent of cefiderocol (CFDC) represents a change of paradigm in the current landscape of antimicrobial stewardship. This siderophore-conjugated cephalosporin is actively taken up by iron transporters overcoming the poor permeability of GNs outer membrane (Table 1). This way, the effect of porin channel mutations and efflux pump upregulation is negligible. Once it has reached the periplasmic space, CFDC maintains structural stability against both serine- and metallo-enzymes, exerting a wide spectrum of activity that comprises ESBL, AmpC, KPC, OXA and MBLs and non-fermentative GN MDROs such as *P. aeruginosa*, *A. baumannii* and *S. maltophilia* [57]. An in vitro study conducted on a collection of 231 selected high-risk GN MDROs showed CFDC to have the strongest activity in respect to comparators. In particular, CFDC was active on all KPC-*Kp*, including those resistant to CZA, on DTR-*Pa*, including those resistant to ceftolozane/tazobactam and on *S. maltophilia*, including those resistant to levofloxacin and trimethoprim/sulfamethoxazole [58]. Lee and colleagues investigated the in vitro susceptibilities of bacteraemic CRE collected in the Taiwanese SMART program. CFDC was the most effective agent, with only 4.5% of resistant isolates, closely followed by CZA that was active on 88.5% of *E. coli* and 93.7% of *K. pneumoniae* [59]. Recently, combined data from SIDERO-WT and SIDERO-Proteeae on 20,911 *Enterobacterales* and non-fermenter isolates collected in 24 European countries displayed CFDC activity against over 97%, regardless of infection site. CFDC had similar activity to colistin against *Acinetobacter spp* (∼90%) and far superior against *S. maltophilia* (99.6% vs. 67.2%). Notably, the drop of activity between carbapenem-susceptible and CR strains of *K. pneumoniae* was deeper for CFDC (98.0% vs. 69.2%) respect to CZA (99.6% vs. 75%), while the opposite effect occurred for *E. coli* strains (CFDC 99.4% vs. 77.8%; CZA 99.7% vs. 33.3%). When considering infections by DTR-*Pa*, the difference was striking, with 97.5% susceptibility to CFDC vs. 44.1% to ceftolozane/tazobactam and 43.2% to CZA, respectively [60]. CFDC was also evaluated against a collection of 150 DTR-*Ab* and was found to be active on 94% of the colistin-resistant isolates [61]. Interestingly, with the iron chelation property of its chlorocatechol sidechain, CFDC was superior to comparators in biofilm reduction of *P. aeruginosa*, *K. pneumoniae*, *S. maltophilia*, *B. cepacia*, *E. coli* and *A. baumannii* [62].

When considering specific carbapenemase type, the SIDERO-CR European isolates showed excellent activity of CFDC on KPC, OXA-48-like and Verona Integron-encoded MBL (VIM). However, effectiveness on NDM-producing *Enterobacterales* was suboptimal [63]. Accordingly, Zalacain et al., when testing the new MBL inhibitor ANT2681 on NDM-producers against comparator agents, found higher effectiveness of a aztreonam/avibactam combination compared to CFDC, especially on *E. coli* NDM producer [64].

Although still rare, some strains with resistance to CFDC have been reported. The mechanisms involved would be mostly represented by mutations in iron transporters. In addition, Hobson et al. pointed out the risk of cross-resistance with ceftazidime is caused by extension of KPC spectrum through specific mutations (namely KPC-31). Furthermore, they revealed a strong impact of a high-inoculum effect on CFDC MICs, which is even more worrisome [65]. Tiseo et al. described a case of recurrent KPC-*Kp* bacteremia successfully treated with MVB after in vivo, CZA-induced resistance development to CZA and CFDC [44]. Moreover, a case of a NDM producing *E. col*i intra-abdominal infection in a hematopoietic stem cell transplant recipient was reported where CFDC resistance evolved after 19 days of administration. Whole genome sequencing analysis indicated a *bla*_NDM-5_ copy number increase that reduced CFDC susceptibility [66].

CFDC was challenged in the APEKS-cUTI trial vs. imipenem-cilastatin for the treatment of cUTI [67] and in the APKES-NP trial in comparison to high-dose, prolonged-infusion meropenem for HAP [68], demonstrating non-inferiority. The CREDIBLE-CR trial compared CFDC with BAT in a CR pathogen-focused trial, as EMA requested for approval [69]. Unluckily, a higher mortality rate in CFDC group, probably driven by *Acinetobacter*, tempered the enthusiasm on this new molecule [70]. It should be noted that patients receiving CFDC had a higher baseline prevalence of moderate and severe renal dysfunction, chronic pulmonary disease, liver impairment and severe infection status (based on clinical judgement), a higher rate of ICU admission, ongoing or recent shock, as well as higher values of CPIS and SOFA score at the time of randomization. Moreover, it appears exceptionally low, and thus poorly comparable, to the mortality rate occurred among patients affected by *Acinetobacter spp.* infections in CREDIBLE-CR and treated with BAT (18%), as it usually exceeds 40% in the literature [71,72].

Real-life experiences with CFDC are limited but slowly expanding. In a case series, three critically ill patients with DTR-*Ab* infection obtained clinical improvement and microbiological eradication [73]. An Italian casuistry included 10 ICU subjects that received compassionate use of CFDC after failure to respond to previous therapy for CR non-fermenting GNs and CRE. Microbiologic failure occurred in two burn patients and clinical failure in one COVID-19 patient [74]. Bavaro et al. presented 13 patients with severe infection by MDRO (mainly CR *Acinetobacter*) and relevant comorbidities including respiratory failure, post-surgical infections and profound immunocompromise. Eradication with no recurrence was achieved in the whole cohort. Interestingly, no patient received CFDC monotherapy. Authors displayed different companion drugs, principally represented by fosfomycin, high-dose tigecycline and colistin [75]. In comparison, in a case series of 13 ICU admitted subjects with DTR-*Ab* infection treated with compassionate CFDC, trough concentration revealed that 23% had a suboptimal *f*C_min_/MIC ratio (i.e., failure to achieve PK/PD target). This group underwent microbiological failure in 80% of the cases [76]. The few reports available in the literature concerning the treatment of MDRO prosthetic joint infections with CFDC obtained encouraging clinical and microbiologic outcomes possibly linked to the peculiar activity of this agent in the biofilm [77,78].

Although data are still limited, combination treatment or intensified dosages will probably be necessary in some critically ill patients with septic shock and/or with high inoculum infections and those caused by the hardest-to-treat pathogens, to maintain and make the best of the extraordinary potential of CFDC. An in vitro study showed a high potency of CFDC against *S. maltophilia*, one of the most challenging MDROs, and synergy was detected when combined with levofloxacin, minocycline, polymyxin B or trimethoprim-sulfamethoxazole [79]. In the study by Abdul-Mutakabbir et al., six strains of a collection of DTR-*Ab* had high baseline MIC to CFDC and whole genome sequencing revealed extended-spectrum AmpC, OXA-51 like and OXA-23 carbapenemase. Authors observed an average 28-fold decline in the CFDC MIC values with the addition of β-lactamase inhibitors tazobactam, sulbactam, avibactam and clavulanic acid. Avibactam produced the strongest reduction. Furthermore, they demonstrated synergistic activity of CFDC with meropenem, amikacin, tigecycline, minocycline and ampicillin/sulbactam in all these isolates [61].

Ultimately, although active towards most of the resistance mechanisms that are currently widespread, CFDC can be considered of choice for infections caused by MBL producing *Enterobacterales* and non-fermenting pathogens with DTR, whereas it could be considered a reasonable alternative to MVB and CZA for treating KPC and OXA-48 producing *Enterobacterales* (Figure 1).

**Table 1:** Principal features of ceftazidime/avibactam, meropenem/vaborbactam and cefiderocol.

Antibiotic	Mechanism of Action	In-Vitro Activity	Emergence of Resistance	Use in Combination	Disadvantages	Comments
Ceftazidime/avibactam	β-lactam/β-lactamase inhibitor Avibactam is a non-β-lactam inhibitor through a reversible mechanism that regenerates an intact molecule of avibactam, allowing for inhibition of further enzymes	-*Enterobacterales* • ESBL • CTX-M • CPE (KPC, OXA-48) -MDR/XDR *P. aeruginosa* -*S. maltophilia* Synergism with carbapenems, tigecycline and fosfomycin against KPC-producing *K. pneumoniae* [80,81] Synergism with fosfomycin against *P. aeruginosa*	Yes (point mutations in the Ω-loop of lactamase; mutations in Omp35 and Omp36 porin channels and efflux pumps overexpression) Risk of in vivo resistance: -CRRT -pneumonia -septic thrombosis -delayed/absence of source control	Used as monotherapy or in combination No definite evidence on superiority of monotherapy over combination therapy In real-life studies, mostly used in combination	-No activity against MBLs -No activity against CRAB -Emergence of resistance	Decreasing susceptibility to this agent during treatment represents a significant concern Combination with aztreonam restores activity against MBL ELF concentration is 30% compared to plasma concentration [42]
Meropenem/vaborbactam	β-lactam/β-lactamase inhibitor Vaborbactam is a competitive inhibitor with a boron-ring structure with high activity towards KPC (K_iapp_ = 69 nM)	-*Enterobacterales* • ESBL • CTX-M • CPE (KPC)	Yes (increased KPC production and porin mutations) Less emergence of resistance than ceftazidime/avibactam	Mostly used as monotherapy	-No activity against MBL -No activity against OXA-48 -No activity against XDR-*P. aeruginosa* -No activity against CRAB	Reduced emergence of resistance as compared to ceftazidime/avibactam Combination with aztreonam restores activity against MBL ELF concentrations are 65% (meropenem) and 79% (vaborbactam) compared to plasma concentration
Cefiderocol	Siderophore-conjugated cephalosporin actively taken up by iron transporter	-*Enterobacterales* • ESBL • CTX-M • CPE (KPC, OXA-48, MBL) -MDR/XDR-Pa -*S. maltophilia* -CRAB -*B. cepaciae*	Yes (mutations in iron transporters) Risk of in vivo resistance due to increased copy numbers of bla NDM genes	Used as monotherapy or in combination	Suboptimal activity against NDM	Combination treatment may probably be necessary in high inoculum infections Due to suboptimal activity against NDM, combination with aztreonam should be considered Anti-biofilm activity

## 3. Algorithm Construction

To preserve the effectiveness of a slowly expanding armamentarium against MDROs and, at the same time, guarantee appropriateness of empirical therapy to critically ill patients, we need a shared criteria for a rational use of new antibiotics. A persuasive, education-based approach of AMS proved to be effective in decreasing antibiotic consumption, incidence and mortality of MDRO BSIs [82]. In this regard, systematic consultation of a dedicated infectious diseases specialist demonstrated the reduction antimicrobial costs, overall hospital and ICU length of stay and mortality [83]. Furthermore, it leads to a more streamlined use of drugs and increased adherence to management guidelines resulting in an advantage for patients in terms of outcome and for community in terms of reduced resistances [84].

In Figure 1 we represented an algorithm to address a suspected infection by a MDRO and perform a reasoned prescription of CZA, MVB and CFDC. The algorithm is discussed below.

### 3.1. Sample Collection, Source Control, Severity of Infection and Risk of Death

After a syndromic framework analysis based on patient clinical appearance and history, the collection of multiple biological samples represents the first step as it provides the chance to characterize the resistance profile we are dealing with. Concurrently, an early source control, following the principles of damage control, is of capital importance to maximize the chances of survival. Endovascular, intrabdominal, soft-tissue and obstructive urinary infection are the most frequent conditions addressing a surgical commitment [85].

Moreover, we deemed that patients in a critical state or with pre-existent conditions posing them at immediate risk of life in case of confirmed MDRO infection constitute the group that would gain the maximum benefit from an early appropriate therapy. The conditions of severe HAP or VAP, septic shock and severe immunodeficiency were taken into considerations for an enlightened prescription of CZA, MVB and CFDC. In addition, the risk of death estimated through the INCREMENT-CPE score [86], validated for CRE only, was included for CZA and MVB. Besides, empirical coverage of gram-positive pathogens is possibly less troublesome but strongly recommended in settings with a high rate of methicillin-resistant *Staphylococcus aureus* (MRSA) (prevalence >20%) and for inpatients with known colonization [87].

When VAP is suspected in a severely ill subject, the initiation of antimicrobial therapy should not be withheld because delayed treatment is associated with increased morbidity and mortality [88]. Furthermore, an inappropriate choice, related to the presence of MDROs unaffected by the empirical treatment, is the most relevant potentially modifiable prognostic factor [89]. Unfortunately, correct diagnosis of VAP is tricky. Clinical Pulmonary Infection Score (CPIS), with six points as cut-off, is a non-invasive diagnostic tool with acceptable accuracy [90], while a reduced PaO2/FiO2 ratio showed a prognostic value in VAP [91].

An observational retrospective study was conducted on 102 adults hospitalized in ICU with KPC-*Kp* BSI: septic shock occurred in 39.2% and infection-related mortality in 42.2% of cohort. Authors found that patients appropriately treated within the first 24 h had significantly lower 30-day mortality and in general the time (in hours) from blood culture collection to administration of in vitro active antibiotic treatment was independently associated with outcome [92]. Consistent findings were observed in a real-world multicenter study with early (within 48 h) MVB administration significantly associated with patient survival [93]. In bloodstream and even nonbacteremic CRE infections, the Pitt score reliably predicted mortality, with ≥4 confirmed as the best cut-off point [94,95]. In recent times, the Pitt score also proved to be an independent predictor of mortality in *P. aeruginosa* [96] and *A. baumannii* [97] BSIs.

Infections by GN MDROs significantly impact outcome in solid organ transplant (SOT) recipients. Wan et al. estimated an incidence between 10% and 75% of MDRO BSI in SOT, with *K. pneumoniae*, *P. aeruginosa* and *A. baumannii* as the most frequent pathogens. Mortality was between 18% and 44% [98]. In patients with chemotherapy-induced neutropenia, bacterial colonization is considered the first step in infection development, followed by translocation of the mucosal barrier. In an observational prospective cohort of adults with haematological malignancies who received chemotherapy and underwent febrile neutropenia, the lowest mortality rate was documented in the non-colonized group [99]. In an Italian casuistry, 46% of patients with a positive rectal swab at the time of stem cell transplantation developed an overt infection. GN MDROs caused a heavy reduction of overall survival and were considered to be the most frequent cause of death in recipients of allogeneic transplant [100]. In high risk KPC-Kp-colonized haematological patients (mainly affected by acute myeloid leukemia) the pre-emptive administration of an active antimicrobial regimen for the empiric treatment of febrile neutropenia resulted in a drop from 67% to 11% of KPC-Kp BSI-related mortality [101].

On the contrary, waiting for a microbiological identification before starting administration of antimicrobials would be appropriate for hemodynamically stable, with no organ damage nor any risk factors for a sudden deterioration (i.e., neutropenia) patients. If the physician still considers it useful the initiation of an empirical antibiotic regimen, the use of molecules with anti-*Pseudomonas* activity (ceftazidime, cefepime, PTZ), possibly associated with anti-MRSA coverage, is probably appropriate. In this regard, ceftobiprole, a fifth generation, recently introduced cephalosporin with anti-MRSA and anti-*Pseudomonas* activity, could represent a reasonable empiric choice in hospital-acquired pneumonia in non-mechanically ventilated patients [102].

### 3.2. Risk Factors, Colonization, and Ecology

Unfortunately, most of the risk factors for MDRO infections are similar between different GN organisms [89,103,104], and not too different for Gram-positives [105] and even fungi [106]. As a matter of fact, severe comorbidities, immunodeficiency, advanced age, invasive procedures, recent exposure to antimicrobials, previous and prolonged hospitalization are common risk factors for almost all healthcare-associated MDRO infections. For this reason, risk factors often provide little or no help to clinicians that must decide on antibiotic treatment empirically.

We assumed patient colonization by a pathogenic MDRO as the starting point to identify subjects at elevated risk of MDRO infection. In fact, contrarily to the redundant nature of risk factors, colonization of a body site with a specific pathogen usually increases the risk of infection by the same colonizing organism [107]. In 2014 Giannella et al. found a KPC-*Kp* BSI incidence rate of 7.8% among KPC-*Kp* rectal carriers. They proposed a score that included admission to ICU, invasive abdominal procedures, chemotherapy/radiation therapy and body site colonization as predictors of KPC-*Kp* BSI development. Of note, colonization at multiple body sites was the strongest predictor of BSI [108]. Later, the INCREMENT-CPE score (ICS) was developed and validated from a multinational retrospective cohort. This tool demonstrated good reliability in predicting early mortality in patients undergoing to CPE BSI [86]. In 2018 Cano et al., in a prospective observational cohort study carried out during a prolonged KPC-*Kp* outbreak, provided external validation to the Giannella risk score (GRS) for the prediction of KPC-*Kp* infection among rectal carriers, with <7 and ≥7 as optimal cut-off. They also validated the ICS for subsequent 30-day mortality, with <8 and ≥8 points associated with low and high risk, respectively. Moreover, authors combined the two scores in a management algorithm to support physicians in deciding which patients should start rapidly empirical treatment and who would benefit from a combinatory regimen [109]. The retrospective study by Tumbarello et al. on patients with KPC-*Kp* infection treated with CZA recently confirmed the impact of INCREMENT-CPR score on mortality [25].

Alongside individual colonization, knowledge of local ecology has been demonstrated to be a helpful tool to forecast the presumable pattern of susceptibility, especially in settings with elevated prevalence of MDROs [88]. In fact, the contamination of the hospital environment with MDROs represents the biggest reservoir for outbreak development and for endemic persistence. If the aqueous environment of the hospital represents the source of MDR *Enterobacterales*, dry surfaces and medical equipment are associated with Gram-positive and non-fermenting GN organisms [110]. Active microbiological surveillance, both focused on inpatient and hospital environments, was shown to be helpful in controlling the onward spread of MDROs and guiding infection control practices [111].

In the algorithm, cumulative data from local ecology and patient colonization indicate the MDRO most likely to be responsible and that should be included in the spectrum of empirical treatment. As explained above, MVB exerts the strongest activity on KPC producers but, at the same time, has the narrowest spectrum on MDROs among the new antimicrobials. CZA provides activity also on OXA-48 producers and on DTR-*Pa*, but suffers the risk of underexposure in some clinical situations. Although many data still need confirmation, CFDC possesses high potency and penetration as well as the wider spectrum on MDROs. Therefore, empirical CFDC might be considered in settings where many different types of resistance mechanisms are widespread (Table 1).

### 3.3. Identification, De-Escalation, and Combination

As elegantly summed up by Timsit et al., culture-based methods remain the gold standard to identify causative pathogens, but rapid molecular alternatives are increasingly deployed, with multiplex-PCR and next-generation sequencing among the most pervasive techniques [85]. At the time of writing, many hospitals have scant availability of such resources. Therefore, diagnostic stewardship programs are being implemented for an evidence-based use of these new fast diagnostic tools that may facilitate the diagnostic process of common infectious syndromes [112]. Obviously, these tests can be considered in our algorithm as a tool for faster access to targeted therapy. Once microbiological identification and characterization are completed, treatment refinement and de-escalation are key components of AMS, with the aim to optimize the spectrum of activity and decrease the emergence of resistance, adverse events and costs [85].

In the algorithm, we suggested definite treatment regimens for the most common resistance patterns assuming demonstrated in vitro susceptibility and taking into account recently updated guidelines [113]. There is a lack of randomized controlled trials for recently introduced antimicrobials, so evidence was almost totally obtained from in vitro and observational studies. Among patients with a BSI from an ESBL producer, carbapenems have demonstrated improved 30-day survival over PTZ [114]. Ceftolozane/tazobactam provides an interesting carbapenem-sparing alternative for ESBL *Enterobacterales*. CZA use may be considered in specific cases, but in general this combination should be preserved for CR infections, as other β-lactam and non-β-lactam options are available for ESBLs [115]. When dealing with a KPC producer, MVB probably represents the preferred choice, but CZA and CFDC are effective alternatives [113]. Literature evidence on the treatment of OXA-48 producing organisms is still scarce. CZA is the preferred choice and CFDC may represent a reasonable alternative [113]. Ceftolozane/tazobactam and CZA demonstrated optimal efficacy for DTR-*Pa* [28] and CFDC represents again another reasonable treatment option [113]. MBL-producing bacteria are an increasingly expanding threat, but treatment possibilities remain an unmet medical need. CFDC is a recommended therapy for MBLs [113]. A Greek study showed combinations of CZA + aztreonam, MVB + aztreonam and imipenem/relebactam + aztreonam to have synergistic in vitro activity against 40 MBL *K. pneumoniae* (mainly NDM) in 97.5%, 72.5% and 97.5%, respectively [116]. To the best of our knowledge, no clinical experience is available for MVB + aztreonam combination, but CZA + aztreonam was recently demonstrated to be a suitable option for the treatment of MBL producers [32]. At present, DTR-*Ab* is probably the pathogen with the most limited available treatment options. In vitro and early clinical evidence showed CFDC to be a promising and effective tool for *Acinetobacter* infections [60,73,74].

The pros and cons of a combinatory antimicrobial regimen in GN MDRO infections are a controversial issue that goes beyond the focus of this work. If the main advantages could be represented by synergy, enlarged spectrum of activity, and limited risk of resistance development, disadvantages include increased adverse events rate (such as *Clostridioides difficilis* infection) and costs [75]. In the meta-analysis by Agyeman et al. on 3352 patients with infection by CR *K. pneumoniae*, monotherapy was associated with higher mortality but no differences in clinical and microbiological outcomes was demonstrated [21]. A recent systematic review stated that, despite the fact that combinations are widely used in real-life (>50% of CZA prescriptions), there is currently not enough evidence to make conclusions whether combo regimens are more effective than monotherapy [117]. In any case, it is interesting to note that in some studies a demonstrated benefit of dual therapy was restricted to the most severely ill patients with high probability of death [85].

We have chosen to incorporate in our place in therapy a role for fosfomycin, an old-fashioned rediscovered agent, which demonstrated an extreme versatility, showing synergism with many old and new antimicrobials against a wide range of MDROs and providing an additional carbapenem-sparing regimen opportunity [118]. Since pneumonia was recognized as a risk factor for CZA-resistance development, some have proposed that fosfomycin, with its excellent lung PK, may compensate this phenomenon [117]. In a 6-year retrospective study on 136 patients with DTR-*Pa* pneumonia, combination therapy, mainly including fosfomycin, was clearly associated with a 28-day survival benefit [119].

## 4. Conclusions

We have proposed an algorithm for a reasonable use of CZA, MVB and CFDC, three new antimicrobial agents on which many hopes have been placed. The notions and reasoning it is composed of are mainly derived from observational analysis, in vitro and pharmacokinetic studies with little evidence from pivotal trials. However, we believe it could represent a useful starting point for decisions in settings where critical patients are involved and a high frequency of MDRO isolations must be taken into account. Future studies in this field will certainly contribute to making it comprehensive and possibly effective.

## References

[1] WHO Website, Newsroom, Antibiotic Resistance 31 July 2020. https://www.who.int/news-room/fact-sheets/detail/antibiotic-resistance.

[2] European Centre for Disease Prevention and Control (2019). Carbapenem-Resistant Enterobacteriaceae. https://www.ecdc.europa.eu/en/publications-data/carbapenem-resistant-Enterobacteriaceae-second-update#no-link.

[3] Haller S., Kramer R., Becker K., Bohnert J.A., Eckmanns T., Hans J.B., Hecht J., Heidecke C.D., Hübner N.O., Kramer A. (2019). Extensively drug-resistant *Klebsiella pneumoniae* ST307 outbreak, north-eastern Germany, June to October 2019. Eur. Surveill..

[4] Cassini A., Högberg L.D., Plachouras D., Quattrocchi A., Hoxha A., Simonsen G.S., Colomb-Cotinat M., Kretzschmar M.E., Devleesschauwer B., Cecchini M. (2019). Attributable deaths and disability-adjusted life-years caused by infections with antibiotic-resistant bacteria in the EU and the European Economic Area in 2015: A population-level modelling analysis. Lancet Infect. Dis..

[5] Cangini A., Fortinguerra F., Di Filippo A., Pierantozzi A., Da Cas R., Villa F., Trotta F., Moro M.L., Gagliotti C. (2021). Monitoring the community use of antibiotics in Italy within the National Action Plan on antimicrobial resistance. Br. J. Clin. Pharmacol..

[6] Tacconelli E., Pezzani M.D. (2019). Public health burden of antimicrobial resistance in Europe. Lancet Infect. Dis..

[7] Kadri S.S., Adjemian J., Lai Y.L., Spaulding A.B., Ricotta E., Prevots D.R., Palmore T.N., Rhee C., Klompas M., Dekker J.P. (2018). National Institutes of Health Antimicrobial Resistance Outcomes Research Initiative (NIH–ARORI). Difficult-to-Treat Resistance in Gram-negative Bacteremia at 173 US Hospitals: Retrospective Cohort Analysis of Prevalence, Predictors, and Outcome of Resistance to All First-line Agents. Clin. Infect. Dis..

[8] Bassetti M., Labate L., Russo C., Vena A., Giacobbe D.R. (2021). Therapeutic options for difficult-to-treat *Acinetobacter baumannii* infections: A 2020 perspective. Expert Opin. Pharmacother..

[9] Mammina C., Palma D.M., Bonura C., Plano M.R.A., Monastero R., Sodano C., Calà C., Tetamo R. (2010). Outbreak of infection with *Klebsiella pneumoniae* sequence type 258 producing *Klebsiella pneumoniae* Carbapenemase 3 in an intensive care unit in Italy. J. Clin. Microbiol..

[10] Bianco A., Quirino A., Giordano M., Marano V., Rizzo C., Liberto M.C., Focà A., Pavia M. (2016). Control of carbapenem-resistant *Acinetobacter baumannii* outbreak in an intensive care unit of a teaching hospital in Southern Italy. BMC Infect. Dis..

[11] Agodi A., Barchitta M., Auxilia F., Brusaferro S., D’Errico M.M., Montagna M.T., Pasquarella C., Tardivo S., Arrigoni C., Fabiani L. (2018). Collaborators. Epidemiology of intensive care unit-acquired sepsis in Italy: Results of the SPIN-UTI network. Ann. Ig..

[12] Moniz P., Coelho L., Póvoa P. (2021). Antimicrobial Stewardship in the Intensive Care Unit: The Role of Biomarkers, Pharmacokinetics, and Pharmacodynamics. Adv. Ther..

[13] Soman R., Bakthavatchalam Y.D., Nadarajan A., Dwarakanathan H.T., Venkatasubramanian R., Veeraraghavan B. (2021). Is it time to move away from polymyxins? Evidence and alternatives. Eur. J. Clin. Microbiol. Infect. Dis..

[14] (2017). WHO Website, News, WHO Publishes List of Bacteria for Which New Antibiotics Are Urgently Needed. https://www.who.int/news/item/27-02-2017-who-publishes-list-of-bacteria-for-which-new-antibiotics-are-urgently-needed.

[15] Doi Y. (2019). Treatment Options for Carbapenem-resistant Gram-negative Bacterial Infections. Clin. Infect. Dis..

[16] van Duin D., Bonomo R.A. (2016). Ceftazidime/Avibactam and Ceftolozane/Tazobactam: Second-generation β-Lactam/β-Lactamase Inhibitor Combinations. Clin. Infect. Dis..

[17] Papp-Wallace K.M., Mack A.R., Taracila M.A., Bonomo R.A. (2020). Resistance to Novel β-Lactam-β-Lactamase Inhibitor Combinations: The “Price of Progress”. Infect. Dis. Clin. North Am..

[18] Shirley M. (2018). Ceftazidime-Avibactam: A Review in the Treatment of Serious Gram-Negative Bacterial Infections. Drugs.

[19] EMA Website, European Medicines Agency: Zavicefta Medicine Overview. https://www.ema.europa.eu/en/medicines/human/EPAR/zavicefta.

[20] Tzouvelekis L.S., Markogiannakis A., Psichogiou M., Tassios P.T., Daikos G.L. (2012). Carbapenemases in *Klebsiella pneumoniae* and other *Enterobacteriaceae*: An evolving crisis of global dimensions. Clin. Microbiol. Rev..

[21] Agyeman A.A., Bergen P.J., Rao G.G., Nation R.L., Landersdorfer C.B. (2020). A systematic review and meta-analysis of treatment outcomes following antibiotic therapy among patients with carbapenem-resistant *Klebsiella pneumoniae* infections. Int. J. Antimicrob. Agents.

[22] Tumbarello M., Trecarichi E.M., Corona A., De Rosa F.G., Bassetti M., Mussini C., Menichetti F., Viscoli C., Campoli C., Venditti M. (2019). Efficacy of Ceftazidime-Avibactam Salvage Therapy in Patients with Infections Caused by *Klebsiella pneumoniae* Carbapenemase-producing K. pneumoniae. Clin. Infect. Dis..

[23] Tsolaki V., Mantzarlis K., Mpakalis A., Malli E., Tsimpoukas F., Tsirogianni A., Papagiannitsis C., Zygoulis P., Papadonta M.E., Petinaki E. (2020). Ceftazidime-Avibactam to Treat Life-Threatening Infections by Carbapenem-Resistant Pathogens in Critically Ill Mechanically Ventilated Patients. Antimicrob. Agents Chemother..

[24] Karaiskos I., Daikos G.L., Gkoufa A., Adamis G., Stefos A., Symbardi S., Chrysos G., Filiou E., Basoulis D., Mouloudi E. (2021). Hellenic Ceftazidime/Avibactam Registry Study Group. Ceftazidime/avibactam in the era of carbapenemase-producing *Klebsiella pneumoniae*: Experience from a national registry study. J. Antimicrob. Chemother..

[25] Tumbarello M., Raffaelli F., Giannella M., Mantengoli E., Mularoni A., Venditti M., De Rosa F.G., Sarmati L., Bassetti M., Brindicci G. (2021). Ceftazidime-avibactam use for KPC-Kp infections: A retrospective observational multicenter study. Clin. Infect. Dis..

[26] Aktaş Z., Kayacan C., Oncul O. (2012). In vitro activity of avibactam (NXL104) in combination with β-lactams against Gram-negative bacteria, including OXA-48 β-lactamase-producing *Klebsiella pneumoniae*. Int. J. Antimicrob. Agents.

[27] De la Calle C., Rodríguez O., Morata L., Marco F., Cardozo C., García-Vidal C., Del Río A., Feher C., Pellicè M., Puerta-Alcalde P. (2019). Clinical characteristics and prognosis of infections caused by OXA-48 carbapenemase-producing *Enterobacteriaceae* in patients treated with ceftazidime-avibactam. Int. J. Antimicrob. Agents.

[28] Hirsch E.B., Brigman H.V., Zucchi P.C., Chen A., Anderson J.C., Eliopoulos G.M., Cheung N., Gilbertsen A., Hunter R.C., Emery C.L. (2020). Ceftolozane-tazobactam and ceftazidime-avibactam activity against β-lactam-resistant *Pseudomonas aeruginosa* and extended-spectrum β-lactamase-producing *Enterobacterales* clinical isolates from U.S. medical centres. J. Glob. Antimicrob. Resist..

[29] Sader H.S., Castanheira M., Duncan L.R., Mendes R.E. (2021). Antimicrobial activities of ceftazidime/avibactam, ceftolozane/tazobactam, imipenem/relebactam, meropenem/vaborbactam, and comparators against *Pseudomonas aeruginosa* from patients with skin and soft tissue infections [published online ahead of print, 2021 Oct 17]. Int. J. Infect. Dis..

[30] Kristóf K., Adámková V., Adler A., Gospodarek-Komkowska E., Rafila A., Billová S., Możejko-Pastewka B., Kiss F. (2021). In vitro activity of ceftazidime-avibactam and comparators against *Enterobacterales* and *Pseudomonas aeruginosa* isolates from Central Europe and Israel, 2014–2017 and 2018. Diagn. Microbiol. Infect. Dis..

[31] (2020). EMA Website. European Medicines Agency. GLOBAL RECALL: Zerbaxa (Ceftolozane/Tazobactam) 1 g/0.5 g Powder for Concentrate for Solution for Infusion. https://www.ema.europa.eu/en/medicines/dhpc/global-recall-zerbaxa-ceftolozane-tazobactam-1-g05-g-powder-concentrate-solution-infusion.

[32] Falcone M., Daikos G.L., Tiseo G., Bassoulis D., Giordano C., Galfo V., Leonildi A., Tagliaferri E., Barnini S., Sani S. (2021). Efficacy of Ceftazidime-avibactam Plus Aztreonam in Patients With Bloodstream Infections Caused by Metallo-β-lactamase-Producing *Enterobacterales*. Clin. Infect. Dis..

[33] Falcone M., Menichetti F., Cattaneo D., Tiseo G., Baldelli S., Galfo V., Leonildi A., Tagliaferri E., Di Paolo A., Pai M.P. (2021). Pragmatic options for dose optimization of ceftazidime/avibactam with aztreonam in complex patients. J. Antimicrob. Chemother..

[34] Lin Q., Zou H., Chen X., Wu M., Ma D., Yu H., Niu S., Huang S. (2021). Avibactam potentiated the activity of both ceftazidime and aztreonam against *S. maltophilia* clinical isolates in vitro. BMC Microbiol..

[35] Wang Y., Wang J., Wang R., Cai Y. (2020). Resistance to ceftazidime-avibactam and underlying mechanisms. J. Glob. Antimicrob. Resist..

[36] Di Bella S., Giacobbe D.R., Maraolo A.E., Viaggi V., Luzzati R., Bassetti M., Luzzaro F., Principe L. (2021). Resistance to ceftazidime/avibactam in infections and colonisations by KPC-producing *Enterobacterales*: A systematic review of observational clinical studies. J. Glob. Antimicrob. Resist..

[37] Carattoli A., Arcari G., Bibbolino G., Sacco F., Tomolillo D., Di Lella F.M., Trancassini M., Faino L., Venditti M., Antonelli G. (2021). Evolutionary Trajectories toward Ceftazidime-Avibactam Resistance in *Klebsiella pneumoniae* Clinical Isolates. Antimicrob. Agents Chemother..

[38] Vena A., Giacobbe D.R., Castaldo N., Cattelan A., Mussini C., Luzzati R., De Rosa F.G., Del Puente F., Mastroianni C.M., Cascio A. (2020). Clinical Experience with Ceftazidime-Avibactam for the Treatment of Infections due to Multidrug-Resistant Gram-Negative Bacteria Other than Carbapenem-Resistant *Enterobacterales*. Antibiotics.

[39] Shields R.K., Nguyen M.H., Chen L., Press E.G., Kreiswirth B.N., Clancy C.J. (2018). Pneumonia and Renal Replacement Therapy Are Risk Factors for Ceftazidime-Avibactam Treatment Failures and Resistance among Patients with Carbapenem-Resistant *Enterobacteriaceae* Infections. Antimicrob. Agents Chemother..

[40] Gatti M., Pea F. (2021). Antimicrobial Dose Reduction in Continuous Renal Replacement Therapy: Myth or Real Need? A Practical Approach for Guiding Dose Optimization of Novel Antibiotics. Clin. Pharmacokinet..

[41] Nicolau D.P., Siew L., Armstrong J., Li J., Edeki T., Learoyd M., Das S. (2015). Phase 1 study assessing the steady-state concentration of ceftazidime and avibactam in plasma and epithelial lining fluid following two dosing regimens. J. Antimicrob. Chemother..

[42] Berkhout J., Melchers M.J., van Mil A.C., Seyedmousavi S., Lagarde C.M., Nichols W.W., Mouton J.W. (2015). Pharmacokinetics and penetration of ceftazidime and avibactam into epithelial lining fluid in thigh- and lung-infected mice. Antimicrob. Agents Chemother..

[43] Oliva A., Curtolo A., Volpicelli L., Cogliati Dezza F., De Angelis M., Cairoli S., Dell’Utri D., Goffredo B.M., Raponi G., Venditti M. (2021). Synergistic Meropenem/Vaborbactam Plus Fosfomycin Treatment of KPC Producing *K. pneumoniae* Septic Thrombosis Unresponsive to Ceftazidime/Avibactam: From the Bench to the Bedside. Antibiotics.

[44] Tiseo G., Falcone M., Leonildi A., Giordano C., Barnini S., Arcari G., Carattoli A., Menichetti F. (2021). Meropenem-Vaborbactam as Salvage Therapy for Ceftazidime-Avibactam-, Cefiderocol-Resistant ST-512 *Klebsiella pneumoniae*-Producing KPC-31, a D179Y Variant of KPC-3. Open Forum Infect. Dis..

[45] Pogue J.M., Bonomo R.A., Kaye K.S. (2019). Ceftazidime/Avibactam, Meropenem/Vaborbactam, or Both? Clinical and Formulary Considerations. Clin. Infect. Dis..

[46] Wenzler E., Scoble P.J. (2020). An Appraisal of the Pharmacokinetic and Pharmacodynamic Properties of Meropenem-Vaborbactam. Infect. Dis. Ther..

[47] Castanheira M., Huband M.D., Mendes R.E., Flamm R.K. (2017). Meropenem-Vaborbactam Tested against Contemporary Gram-Negative Isolates Collected Worldwide during 2014, Including Carbapenem-Resistant, KPC-Producing, Multidrug-Resistant, and Extensively Drug-Resistant *Enterobacteriaceae*. Antimicrob. Agents Chemother..

[48] Langley G.W., Cain R., Tyrrell J.M., Hinchliffe P., Calvopiña K., Tooke C.L., Widlake E., Dowson C.G., Spencer J., Walsh T.R. (2019). Profiling interactions of vaborbactam with metallo-β-lactamases. Bioorg. Med. Chem. Lett..

[49] Hackel M.A., Lomovskaya O., Dudley M.N., Karlowsky J.A., Sahm D.F. (2017). In Vitro Activity of Meropenem-Vaborbactam against Clinical Isolates of KPC-Positive *Enterobacteriaceae*. Antimicrob. Agents Chemother..

[50] Kaye K.S., Bhowmick T., Metallidis S., Bleasdale S.C., Sagan O.S., Stus V., Vazquex J., Zaitsev V., Bidair M., Chorvat E. (2018). Effect of Meropenem-Vaborbactam vs Piperacillin-Tazobactam on Clinical Cure or Improvement and Microbial Eradication in Complicated Urinary Tract Infection: The TANGO I Randomized Clinical Trial. JAMA.

[51] Wunderink R.G., Giamarellos-Bourboulis E.J., Rahav G., Mathers A.J., Bassetti M., Vazquez J., Cornely O.A., Solomkin J., Bhowmick T., Bishara J. (2018). Effect and Safety of Meropenem-Vaborbactam versus Best-Available Therapy in Patients with Carbapenem-Resistant *Enterobacteriaceae* Infections: The TANGO II Randomized Clinical Trial. Infect. Dis. Ther..

[52] Bassetti M., Giacobbe D.R., Patel N., Tillotson G., Massey J. (2019). Efficacy and Safety of Meropenem-Vaborbactam versus Best Available Therapy for the Treatment of Carbapenem-Resistant *Enterobacteriaceae* Infections in Patients without Prior Antimicrobial Failure: A Post Hoc Analysis. Adv. Ther..

[53] EMA Website, European Medicines Agency: Vaborem Medicine Overview. https://www.ema.europa.eu/en/documents/assessment-report/vabomere-epar-public-assessment-report_en.pdf.

[54] Sun D., Rubio-Aparicio D., Nelson K., Dudley M.N., Lomovskaya O. (2017). Meropenem-Vaborbactam Resistance Selection, Resistance Prevention, and Molecular Mechanisms in Mutants of KPC-Producing *Klebsiella pneumoniae*. Antimicrob. Agents Chemother..

[55] Ackley R., Roshdy D., Meredith J., Minor S., Anderson W.E., Capraro G.A., Polk C. (2020). Meropenem-Vaborbactam versus Ceftazidime-Avibactam for Treatment of Carbapenem-Resistant *Enterobacteriaceae* Infections. Antimicrob. Agents Chemother..

[56] Bhowmick T., Weinstein M.P. (2020). Microbiology of Meropenem-Vaborbactam: A Novel Carbapenem Beta-Lactamase Inhibitor Combination for Carbapenem-Resistant *Enterobacterales* Infections. Infect. Dis. Ther..

[57] Sato T., Yamawaki K. (2019). Cefiderocol: Discovery, Chemistry, and In Vivo Profiles of a Novel Siderophore Cephalosporin. Clin. Infect. Dis..

[58] Delgado-Valverde M., Conejo M.D.C., Serrano L., Fernández-Cuenca F., Pascual Á. (2020). Activity of cefiderocol against high-risk clones of multidrug-resistant *Enterobacterales*, *Acinetobacter baumannii*, *Pseudomonas aeruginosa* and *Stenotrophomonas maltophilia*. J. Antimicrob. Chemother..

[59] Lee Y.L., Ko W.C., Lee W.S., Lu P.L., Chen Y.H., Cheng S.H., Lu M.C., Lin C.Y., Wu T.S., Yen M.Y. (2021). In-vitro activity of cefiderocol, cefepime/zidebactam, cefepime/enmetazobactam, omadacycline, eravacycline and other comparative agents against carbapenem-nonsusceptible *Enterobacterales*: Results from the Surveillance of Multicenter Antimicrobial Resistance in Taiwan (SMART) in 2017–2020. Int. J. Antimicrob. Agents.

[60] Candel F.J., Henriksen A.S., Longshaw C., Yamano Y., Oliver A. In vitro activity of the novel siderophore cephalosporin, cefiderocol, in Gram-negative pathogens in Europe by site of infection. Clin. Microbiol. Infect..

[61] Abdul-Mutakabbir J.C., Nguyen L., Maassen P.T., Stamper K.C., Kebriaei R., Kaye K.S., Castanheira M., Rybak M.J. (2021). In Vitro Antibacterial Activity of Cefiderocol against Multidrug-Resistant *Acinetobacter baumannii*. Antimicrob. Agents Chemother..

[62] Pybus C.A., Felder-Scott C., Obuekwe V., Greenberg D.E. (2021). Cefiderocol Retains Antibiofilm Activity in Multidrug-Resistant Gram-Negative Pathogens. Antimicrob. Agents Chemother..

[63] Longshaw C., Manissero D., Tsuji M., Echols R., Yamano Y. (2020). In vitro activity of the siderophore cephalosporin, cefiderocol, against molecularly characterized, carbapenem-non-susceptible Gram-negative bacteria from Europe. JAC Antimicrob. Resist..

[64] Zalacain M., Lozano C., Llanos A., Sprynski N., Valmont T., De Piano C., Davies D., Leiris S., Sable C., Ledoux A. (2021). Novel Specific Metallo-β-Lactamase Inhibitor ANT2681 Restores Meropenem Activity to Clinically Effective Levels against NDM-Positive *Enterobacterales*. Antimicrob. Agents Chemother..

[65] Hobson C.A., Cointe A., Jacquier H., Choudhury A., Magnan M., Courroux C., Tenaillon O., Bonacorsi S., Birgy A. (2021). Cross-resistance to cefiderocol and ceftazidime-avibactam in KPC β-lactamase mutants and the inoculum effect. Clin. Microbiol. Infect..

[66] Simner P.J., Mostafa H.H., Bergman Y., Ante M., Tekle T., Adebayo A., Beisken S., Dzintars K., Tamma P.D. (2021). Progressive Development of Cefiderocol Resistance in Escherichia coli During Therapy Is Associated with Increased blaNDM-5 Copy Number and Gene Expression. Clin. Infect. Dis..

[67] Portsmouth S., van Veenhuyzen D., Echols R., Machida M., Ferreira J.C.A., Ariyasu M., Tenke P., Nagata T.D. (2018). Cefiderocol versus imipenem-cilastatin for the treatment of complicated urinary tract infections caused by Gram-negative uropathogens: A phase 2, randomised, double-blind, non-inferiority trial. Lancet Infect. Dis..

[68] Wunderink R.G., Matsunaga Y., Ariyasu M., Clevenbergh P., Echols R., Kaye K.S., Kollef M., Menon A., Pogue J.M., Shorr A.F. (2021). Cefiderocol versus high-dose, extended-infusion meropenem for the treatment of Gram-negative nosocomial pneumonia (APEKS-NP): A randomised, double-blind, phase 3, non-inferiority trial. Lancet Infect. Dis..

[69] Bassetti M., Echols R., Matsunaga Y., Ariyasu M., Doi Y., Ferrer R., Lodise T.P., Naas T., Niki Y., Paterson D.L. (2021). Efficacy and safety of cefiderocol or best available therapy for the treatment of serious infections caused by carbapenem-resistant Gram-negative bacteria (CREDIBLE-CR): A randomised, open-label, multicentre, pathogen-focused, descriptive, phase 3 trial. Lancet Infect. Dis..

[70] Heil E.L., Tamma P.D. (2021). Cefiderocol: The Trojan horse has arrived but will Troy fall?. Lancet Infect. Dis..

[71] Dickstein Y., Lellouche J., Ben Dalak Amar M., Schwartz D., Nutman A., Daitch V., Yahav D., Leibovici L., Skiada A., Antoniadou A. (2019). AIDA Study Group. Treatment Outcomes of Colistin- and Carbapenem-resistant *Acinetobacter baumannii* Infections: An Exploratory Subgroup Analysis of a Randomized Clinical Trial. Clin. Infect. Dis..

[72] Piperaki E.T., Tzouvelekis L.S., Miriagou V., Daikos G.L. (2019). Carbapenem-resistant *Acinetobacter baumannii*: In pursuit of an effective treatment. Clin. Microbiol. Infect..

[73] Oliva A., Ceccarelli G., De Angelis M., Sacco F., Miele M.C., Mastroianni C.M., Venditti M. (2020). Cefiderocol for compassionate use in the treatment of complicated infections caused by extensively and pan-resistant *Acinetobacter baumannii*. J. Glob. Antimicrob. Resist..

[74] Falcone M., Tiseo G., Nicastro M., Leonildi A., Vecchione A., Casella C., Forfori F., Malacarne P., Guarracino F., Barnini S. (2021). Cefiderocol as Rescue Therapy for *Acinetobacter baumannii* and Other Carbapenem-resistant Gram-negative Infections in Intensive Care Unit Patients. Clin. Infect. Dis..

[75] Bavaro D.F., Belati A., Diella L., Stufano M., Romanelli F., Scalone L., Stolfa S., Ronga L., Maurmo L., Dell’Aera M. (2021). Cefiderocol-Based Combination Therapy for “Difficult-to-Treat” Gram-Negative Severe Infections: Real-Life Case Series and Future Perspectives. Antibiotics.

[76] Gatti M., Bartoletti M., Cojutti P.G., Gaibani P., Conti M., Giannella M., Viale P., Pea F. (2021). A descriptive case series of PK/PD target attainment and microbiological outcome in critically ill patients with documented severe XDR *Acinetobacter baumannii* BSI and/or VAP treated with cefiderocol. J. Glob. Antimicrob. Resist..

[77] Siméon S., Dortet L., Bouchand F., Roux A.L., Bonnin R.A., Duran C., Decousser J.W., Bessis S., Davido B., Sorriaux G. (2020). Compassionate Use of Cefiderocol to Treat a Case of Prosthetic Joint Infection Due to Extensively Drug-Resistant Enterobacter hormaechei. Microorganisms.

[78] Mabayoje D.A., NicFhogartaigh C., Cherian B.P., Tan M.G.M., Wareham D.W. (2021). Compassionate use of cefiderocol for carbapenem-resistant *Acinetobacter baumannii* prosthetic joint infection. JAC Antimicrob. Resist..

[79] Biagi M., Vialichka A., Jurkovic M., Wu T., Shajee A., Lee M., Patel S., Mendes R.E., Wenzler E. (2020). Activity of Cefiderocol Alone and in Combination with Levofloxacin, Minocycline, Polymyxin B, or Trimethoprim-Sulfamethoxazole against Multidrug-Resistant *Stenotrophomonas maltophilia*. Antimicrob. Agents Chemother..

[80] Gaibani P., Lewis R.E., Volpe S.L., Giannella M., Campoli C., Landini M.P., Viale P., Re M.C., Ambretti S. (2017). In vitro interaction of ceftazidime-avibactam in combination with different antimicrobials against KPC-producing *Klebsiella pneumoniae* clinical isolates. Int. J. Infect. Dis..

[81] Romanelli F., De Robertis A., Carone G., Dalfino L., Stufano M., Del Prete R., Mosca A. (2020). In Vitro Activity of Ceftazidime/Avibactam Alone and in Combination with Fosfomycin and Carbapenems Against KPC-producing *Klebsiella pneumoniae*. New Microbiol..

[82] Molina J., Peñalva G., Gil-Navarro M.V., Praena J., Lepe J.A., Pérez-Moreno M.A., Ferràndiz C., Aldabò T., Aguilar M., Olbrich P. (2017). PRIOAM team. Long-Term Impact of an Educational Antimicrobial Stewardship Program on Hospital-Acquired Candidemia and Multidrug-Resistant Bloodstream Infections: A Quasi-Experimental Study of Interrupted Time-Series Analysis. Clin. Infect. Dis..

[83] Butt A.A., Al Kaabi N., Saifuddin M., Krishnanreddy K.M., Khan M., Jasim W.H., Khan T., Sara M., Pitout M., Weber S. (2015). Impact of Infectious Diseases Team Consultation on Antimicrobial Use, Length of Stay and Mortality. Am. J. Med. Sci..

[84] Jiménez-Aguilar P., López-Cortés L.E., Rodríguez-Baño J. (2019). Impact of infectious diseases consultation on the outcome of patients with bacteraemia. Ther. Adv. Infect. Dis..

[85] Timsit J.F., Ruppé E., Barbier F., Tabah A., Bassetti M. (2020). Bloodstream infections in critically ill patients: An expert statement. Intensive Care Med..

[86] Gutiérrez-Gutiérrez B., Salamanca E., de Cueto M., Hsueh P.R., Viale P., Paño-Pardo J.R., Venditti M., Tumbarello M., Daikos G., Pintado V. (2016). Investigators from the REIPI/ESGBIS/INCREMENT Group. A Predictive Model of Mortality in Patients with Bloodstream Infections due to Carbapenemase-Producing *Enterobacteriaceae*. Mayo Clin. Proc..

[87] Lee A.S., de Lencastre H., Garau J., Kluytmans J., Malhotra-Kumar S., Peschel A., Harbarth S. (2018). Methicillin-resistant Staphylococcus aureus. Nat. Rev. Dis. Primers.

[88] Garnacho-Montero J., Corcia-Palomo Y., Amaya-Villar R., Martin-Villen L. (2014). How to treat VAP due to MDR pathogens in ICU patients. BMC Infect. Dis..

[89] Zaragoza R., Vidal-Cortés P., Aguilar G., Borges M., Diaz E., Ferrer R., Maseda E., Nieto M., Nuvials F.X., Ramirez P. (2020). Update of the treatment of nosocomial pneumonia in the ICU. Crit. Care.

[90] Fernando S.M., Tran A., Cheng W., Klompas M., Kyeremanteng K., Mehta S., English S.W., Muscedere J., Cook D.J., Torres A. (2020). Diagnosis of ventilator-associated pneumonia in critically ill adult patients-a systematic review and meta-analysis. Intensive Care Med..

[91] Karakuzu Z., Iscimen R., Akalin H., Kelebek Girgin N., Kahveci F., Sinirtas M. (2018). Prognostic Risk Factors in Ventilator-Associated Pneumonia. Med. Sci. Monit..

[92] Falcone M., Bassetti M., Tiseo G., Giordano C., Nencini E., Russo A., Graziano E., Tagliaferri E., Leonildi A., Barnini S. (2020). Time to appropriate antibiotic therapy is a predictor of outcome in patients with bloodstream infection caused by KPC-producing *Klebsiella pneumoniae*. Crit. Care.

[93] Alosaimy S., Lagnf A.M., Morrisette T., Scipione M.R., Zhao J.J., Jorgensen S.C.J., Mynatt R., Carlson T.J., Jo J., Garey K.W. (2021). Real-world, Multicenter Experience With Meropenem-Vaborbactam for Gram-Negative Bacterial Infections Including Carbapenem-Resistant *Enterobacterales* and *Pseudomonas aeruginosa*. Open Forum Infect. Dis..

[94] Henderson H., Luterbach C.L., Cober E., Richter S.S., Salata R.A., Kalayjian R.C., Watkins R.R., Doi Y., Kaye K.S., Evans S. (2020). The Pitt Bacteremia Score Predicts Mortality in Nonbacteremic Infections. Clin. Infect. Dis..

[95] Al-Hasan M.N., Baddour L.M. (2020). Resilience of the Pitt Bacteremia Score: 3 Decades and Counting. Clin. Infect. Dis..

[96] Recio R., Mancheño M., Viedma E., Villa J., Orellana M.Á., Lora-Tamayo J., Chaves F. (2020). Predictors of Mortality in Bloodstream Infections Caused by *Pseudomonas aeruginosa* and Impact of Antimicrobial Resistance and Bacterial Virulence. Antimicrob. Agents Chemother..

[97] Gu Y., Jiang Y., Zhang W., Yu Y., He X., Tao J., Hou X., Wang H., Deng M., Zhou M. (2021). Risk factors and outcomes of bloodstream infections caused by *Acinetobacter baumannii*: A case-control study. Diagn. Microbiol. Infect. Dis..

[98] Wan Q.Q., Ye Q.F., Yuan H. (2015). Multidrug-resistant Gram-negative bacteria in solid organ transplant recipients with bacteremias. Eur. J. Clin. Microbiol. Infect. Dis..

[99] Alrstom A., Alsuliman T., Daher N., Abouharb R. (2021). The Impact of Modifying Empirical Antibiotic Therapy Based on Intestinal Colonization Status on Clinical Outcomes of Febrile Neutropenic Patients. Infect. Chemother..

[100] Patriarca F., Cigana C., Massimo D., Lazzarotto D., Geromin A., Isola M., Battista M.L., Medeot M., Cerno M., Sperotto A. (2017). Risk Factors and Outcomes of Infections by Multidrug-Resistant Gram-Negative Bacteria in Patients Undergoing Hematopoietic Stem Cell Transplantation. Biol. Blood Marrow Transplant..

[101] Micozzi A., Gentile G., Santilli S., Minotti C., Capria S., Moleti M.L., Barberi W., Cartoni C., Trisolini S.M., Testi A.M. (2021). Reduced mortality from KPC-*K. pneumoniae* bloodstream infection in high-risk patients with hematological malignancies colonized by KPC-*K. pneumoniae*. BMC Infect. Dis..

[102] Giacobbe D.R., De Rosa F.G., Del Bono V., Grossi P.A., Pea F., Petrosillo N., Rossolini G.M., Tascini C., Tumbarello M., Viale P. (2019). Ceftobiprole: Drug evaluation and place in therapy. Expert Rev. Anti Infect. Ther..

[103] Burillo A., Muñoz P., Bouza E. (2019). Risk stratification for multidrug-resistant Gram-negative infections in ICU patients. Curr. Opin. Infect. Dis..

[104] Leal H.F., Azevedo J., Silva G.E.O., Amorim A.M.L., de Roma L.R.C., Palmeira Arraes A.C., Lins Gouveia E., Reis M.G., Mendes A.V., de Oliveira Silva M. (2019). Bloodstream infections caused by multidrug-resistant gram-negative bacteria: Epidemiological, clinical, and microbiological features. BMC Infect. Dis..

[105] Catry B., Latour K., Jans B., Vandendriessche S., Preal R., Mertens K., Denis O. (2014). Risk factors for methicillin resistant *Staphylococcus aureus*: A multi-laboratory study. PLoS ONE.

[106] Keighley C.L., Pope A., Marriott D.J.E., Chapman B., Bak N., Daveson K., Hajkowicz K., Halliday C., Kennedy K., Kidd S. (2021). Risk factors for candidaemia: A prospective multi-centre case-control study. Mycoses.

[107] Detsis M., Karanika S., Mylonakis E. (2017). ICU Acquisition Rate, Risk Factors, and Clinical Significance of Digestive Tract Colonization with Extended-Spectrum Beta-Lactamase-Producing *Enterobacteriaceae*: A Systematic Review and Meta-Analysis. Crit. Care Med..

[108] Giannella M., Trecarichi E.M., De Rosa F.G., Del Bono V., Bassetti M., Lewis R.E., Losito A.R., Corcione S., Saffioti C., Bartoletti M. (2014). Risk factors for carbapenem-resistant *Klebsiella pneumoniae* bloodstream infection among rectal carriers: A prospective observational multicentre study. Clin. Microbiol. Infect..

[109] Cano A., Gutiérrez-Gutiérrez B., Machuca I., Gracia-Ahufinger I., Perèz-Nadales E., Causse M., Castòn J.J., Guzman-Puche J., Torre-Gimenèz J., Kindelàn L. (2018). Risks of Infection and Mortality among Patients Colonized with *Klebsiella pneumoniae* Carbapenemase-Producing *K. pneumoniae*: Validation of Scores and Proposal for Management. Clin. Infect. Dis..

[110] Chia P.Y., Sengupta S., Kukreja A., Ponnampalavanar S.S., Ng O.T., Marimuthu K. (2020). The role of hospital environment in transmissions of multidrug-resistant gram-negative organisms. Antimicrob. Resist. Infect. Control..

[111] Migliara G., Di Paolo C., Barbato D., Baccolini V., Salerno C., Nardi A., Alessandri F., Giordano A., Tufi D., Marinelli L. (2019). Multimodal surveillance of healthcare associated infections in an intensive care unit of a large teaching hospital. Ann. Ig..

[112] Tiseo G., Arena F., Borrè S., Campanile F., Falcone M., Mussini C., Pea F., Sganga G., Stefani S., Venditti M. (2021). Diagnostic stewardship based on patient profiles: Differential approaches in acute versus chronic infectious syndromes. Expert Rev. Anti Infect. Ther..

[113] Tamma P.D., Aitken S.L., Bonomo R.A., Mathers A.J., van Duin D., Clancy C.J. (2021). Infectious Diseases Society of America Guidance on the Treatment of Extended-Spectrum β-lactamase Producing *Enterobacterales* (ESBL-E), Carbapenem-Resistant *Enterobacterales* (CRE), and *Pseudomonas aeruginosa* with Difficult-to-Treat Resistance (DTR-*P. aeruginosa*). Clin. Infect. Dis..

[114] Harris P.N.A., Tambyah P.A., Lye D.C., Mo Y., Lee T.H., Yilmaz M., Alenazi T.H., Arabi Y., Falcone M., Bassetti M. (2018). MERINO Trial Investigators and the Australasian Society for Infectious Disease Clinical Research Network (ASID-CRN). Effect of Piperacillin-Tazobactam vs. Meropenem on 30-Day Mortality for Patients with E coli or *Klebsiella pneumoniae* Bloodstream Infection and Ceftriaxone Resistance: A Randomized Clinical Trial. JAMA.

[115] Karaiskos I., Giamarellou H. (2020). Carbapenem-Sparing Strategies for ESBL Producers: When and How. Antibiotics.

[116] Maraki S., Mavromanolaki V.E., Moraitis P., Stafylaki D., Kasimati A., Magkafouraki E., Scoulica E. (2021). Ceftazidime-avibactam, meropenen-vaborbactam, and imipenem-relebactam in combination with aztreonam against multidrug-resistant, metallo-β-lactamase-producing *Klebsiella pneumoniae*. Eur. J. Clin. Microbiol. Infect. Dis..

[117] Meini S., Viaggi B., Tascini C. (2021). Mono vs. combo regimens with novel beta-lactam/beta-lactamase inhibitor combinations for the treatment of infections due to carbapenemase-producing *Enterobacterales*: Insights from the literature. Infection.

[118] Horcajada J.P., Montero M., Oliver A., Sorlí L., Luque S., Gómez-Zorrilla S., Benito N., Grau S. (2019). Epidemiology and Treatment of Multidrug-Resistant and Extensively Drug-Resistant *Pseudomonas aeruginosa* Infections. Clin. Microbiol. Rev..

[119] Khawcharoenporn T., Chuncharunee A., Maluangnon C., Taweesakulvashra T., Tiamsak P. (2018). Active monotherapy and combination therapy for extensively drug-resistant *Pseudomonas aeruginosa* pneumonia. Int. J. Antimicrob. Agents.

